# Supplementary material for: High CSF thrombin concentration and activity is associated with an unfavorable outcome in patients with intracerebral hemorrhage
Source: PLoS One. 2020 Nov 11;15(11):e0241565. doi: 10.1371/journal.pone.0241565 (PMC7657554; doi:10.1371/journal.pone.0241565)
Supplement: S1 Table — (DOCX) [file pone.0241565.s002.docx]

| **S1 Table**: Underlying conditions | | |
| --- | --- | --- |
|  | Number | % / cohort |
| Arterial Hypertension | 15 | 75 |
| Heart disease | 7 | 35 |
| Malignancy | 6 | 30 |
| Oral Anticoagulation | 5 | 25 |
| Previous ICH or Ischemia | 5 | 25 |
| Lung disease | 5 | 25 |
| Substance abuse | 4 | 20 |
| Diverticulitis | 2 | 10 |
| Psychiatric disease | 2 | 10 |
| Diabetes | 2 | 10 |
| Renal failure | 1 | 5 |
| Sarcoidosis | 1 | 5 |
| Chronic infectious disease | 1 | 5 |
| Multiple Sclerosis | 1 | 5 |
| Parkinson’s disease | 1 | 5 |
|  |  |  |
